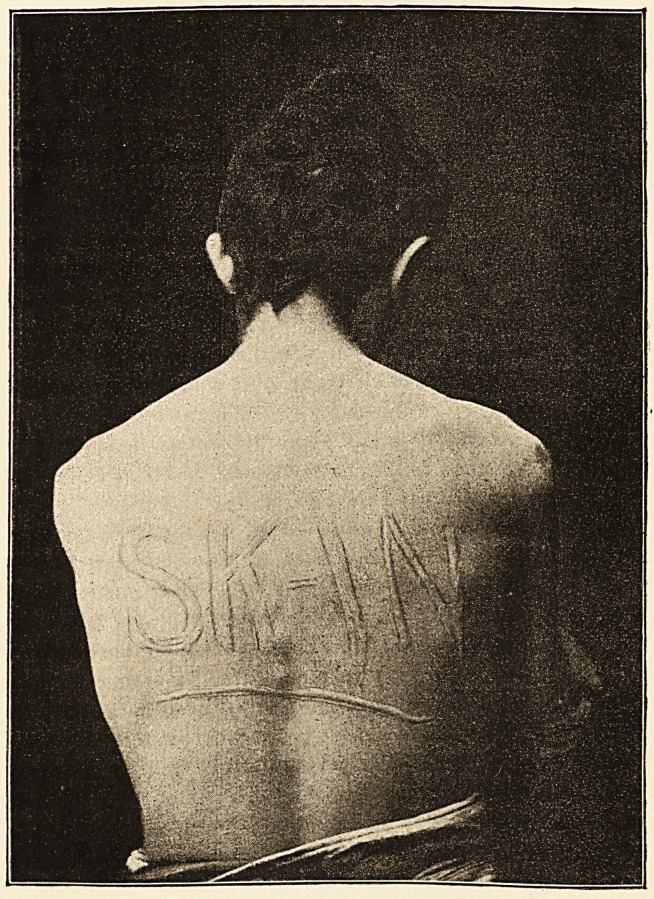# A Case of Factitious Urticaria
*Read before the Bristol Medico-Chirurgical Society.


**Published:** 1890-03

**Authors:** Henry Waldo

**Affiliations:** Physician to the Bristol Royal Infirmary, and in charge of the Department for Skin Diseases


### Clinical Records.

---

A CASE OF FACTITIOUS URTICARIA.* By HENRY WALDO, M.D. Aberd., M.R.C.P. Lond.; Physician to the Bristol Royal Infirmary, and in charge of the Department for Skin Diseases.

The subject of this condition is a male patient, a confectioner, æt. 29. There is such excessive irritability of the cutaneous nerves, that wheals can be excited by local irritation. If letters are inscribed with any bluntly-pointed instrument, white letters with pink borders stand out in bold relief on the skin. (See Engraving over.)

Patient is the subject of acute dyspepsia; he says he has so much pain in his chest at times, that if in bed he is obliged to get up and walk about.

His tongue is coated and relaxed, and the bowels confined. In his business he is subject to sudden alterations of temperature. He complains of a constant intolerable itching all over the skin.

The etiology of this affection is much the same as the etiology of ordinary urticaria, and may be produced by insect bites, or by any kind of external irritation. Many articles of food, by causing irritation indirectly, and medicines of many kinds act in the same way. There is often a predisposing idiosyncrasy on the part of the patient. The gouty diathesis is considered to be a predisposing cause, and there is often defective digestion habitually present. As regards emotional conditions, there is reported the case of a lady in whom the advent

* Read before the Bristol Medico-Chirurgical Society.

SKIN

of strangers produced urticaria; and this sensitiveness increased, until a knock or ring at the front door would determine an immediate outbreak. Urticaria is frequent in jaundice; it is not unusual in albuminuria and glycosuria; and it has been found in association with rheumatism, purpura, and in intermittent fever.

Everything in urticaria points to its being primarily a vaso-motor disturbance, direct or reflex, central or peripheral. The course of events is probably this: A spasmodic contraction is followed by paralytic dilatation of the vessels, and stasis or retardation of the circulation in the papillary layer. Serous exudation then ensues, producing acute œdema, which lifts up the epidermis into a wheal, which is pink at first; but as the fluid increases, the blood is pressed out at the centre, which becomes white, while the periphery is all the more hyperæmic. Whether the muscles of the skin take part in the process is doubtful; but it is supposed by many that they, by their contraction, limit the œdema and increase the prominence of the wheal.

Unna thinks the wheal is produced by a spasm of the large veins of the skin, which normally serve to carry off the lymph.

This patient has been treated with stomach sedatives, and stomachics with saline aperients, and dieted; and he is very much better in himself, and his skin is much less irritable, but the artificial urticaria is still easily produced. He has also taken strophanthus and cannabis indica. He has found benefit from the local application of carbolic acid, dusting powders containing camphor, and liq. carbonis detergens, diluted. The neurotics, belladonna and atropia, which act on the vaso-motor centres, are necessary in treating some of these cases.

## Figures and Tables

**Figure f1:**